# Paper on a Singular Description of Disease Which Appeared in the Island of St. Christopher, in the Latter End of the Year 1827 and Beginning of 1828

**Published:** 1828-07

**Authors:** John Squaer

**Affiliations:** Assistant Surgeon 93d Regiment.


### RHEUMATISMUS FEBRILIS.

*Paper on a singular Description of Disease which appeared in the Island of St. Christopher, in the latter end of the year* 1827 *and beginning of* 1828. By JOHN SQUAER, Esq. assistant Surgeon 93d Regiment.

THE disease which forms the subject of the present paper had broken out for several weeks in the islands in our immediate neighbourhood to leeward, which are all foreign and "free ports," with the exception of the small island of Tortola, (which is a dependency of St. Kitt's,) and have frequent intercourse with us, by means of small English trading vessels; consequently it is not to be wondered at, the facility with which disease of an epidemic nature finds its way here.

It is not meant that the present disease should be considered as having been brought to this island by means of communication: proof enough will be afforded to upset this idea in speaking of the comparative prevalence of this disease amongst the troops of the garrison and inhabitants, even although it is characterised in the newspapers of the island by the title of "*an epidemic of the most painful nature, which the oldest inhabitant did not remember to have seen or heard of before.*"

This disease is classed as a species of rheumatism, in the Quarterly Return of Sick of this garrison, and is styled by me Rheumatismus Febrilis; commonly known by the name of rheumatic fever in this and the neighbouring islands.

In the latter end of December and beginning of January of the present year, many people began to complain of very violent headache, severe pain in the temples, shooting towards the forehead; frequently it was situated in the back of the head, stretching towards the neck and shoulders, which was one of its most painful positions, as the least motion created great agony, and it was difficult to find an easy posture for the head. There frequently was a painful sensation as if the head were drawn down towards one side or another; pain, or, at all events, a disagreeable sense of stiffness, was felt in the eyes, especially when moving them from side to side, or raising them upwards: the patients expressed it, by saying the socket felt as if it were too small for the eyeball; frequently the eyes felt painful to the touch; the adnata was slightly tinged with red vessels.

Shooting pains were at the same time felt in the back, loins, and thighs, particularly immediately over the knees, which soon became fixed and uncommonly severe: the same thing took place in the arms, forearms, wrists, fingers, knees, ankles, and feet, causing lameness; the calves of the legs were similarly affected.

A roseolar eruption came out early in the disease, which covered the wrists and extended up the forearm: it spread over the backs of the hands; the ankles and feet were in the same state; it was sometimes elevated in large wheals, and, when it affected the neck, it was extremely painful: the hands and feet were considerably swelled.

In delicate females, the roseola came out on the face in patches, and on different parts of the body, and remained for several weeks after the other symptoms had disappeared.

It need hardly be added, that motion of any kind greatly aggravated the symptoms, and the gentle pressure of the hand could scarcely be suffered.*

Fever came on simultaneously with these various affections, or very soon was observed in conjunction with them, marked by a sense of heaviness in the head and great listlessness, nausea and loss of appetite, and, in delicate people, the irritability of stomach was sometimes distressing. Severe rigors, and alternating flushes of heat; face flushed; quick full pulse; and hot dry skin; with, in a few cases, delirium, were also observed.

Pain of stomach, sensible to gentle pressure, was present in one or two instances.

The violence of the symptoms and fever lasted from four to five days; but it was never under seven or eight days that all the pains were gone. In most cases, the pains were felt for a much longer time; and in severe attacks, pain and tenderness to the touch remained in the eyes, hands, calves of the legs, ankles, and feet, for weeks afterwards.

These symptoms varied in number and degree of violence, according to circumstances, and were much influenced by mode of living and constitution, sex, and age.

The soldiers composing the garrison of Brimstone Hill were less liable to this disease than the inhabitants; and their attacks were not of so long continuance, nor generally so severe. Nearly all the officers had it, and it was severe in one instance only. The very general run it took amongst the inhabitants had the effect of its being supposed to be epidemic; in many instances, not leaving a family till every one had been attacked.

The young and robust had smart attacks, and fever of shorter duration, and they did not so often labour under its effects; and were even exempt from one or two symptoms that afflicted people of an opposite description.

Delicate females and aged persons had more protracted attacks, and they suffered more from irritability of stomach; and the roseolar eruption in them was most remarkable; and the feet continued swelled and tender, producing lameness for some time: the fingers were also swelled and painful.

On account of the lingering nature of the disease, many were induced to suppose that, during the space of eight or ten weeks, they had fresh attacks, and were even impressed

* The stiffened form, occasioned by the pains in the head connected with the shoulder, and the dread of motion, obtained for it the fantastic name of "the dandy."

with the idea that they must have a third attack before they could get well: this was owing to exposure to the cold damp weather, which at first caused it, and consequently easily re-excited the pains they had not entirely got quit of.

This disease, in all the instances I have witnessed, was considered of a simple, and though of a violent nature, yet there was nothing dangerous in it. It has been said to have terminated fatally in one or two instances in this island: in some of the others, it has caused death in several instances.

This circumstance I am inclined to attribute to some untoward combination of disease, or might be the result of accident, as was the case in one instance. A coloured man, of the town of Old Road, having had symptoms of the disease, thought himself sufficiently well even to go to his work, imprudently bathed in the river, which aggravated the disease to such a degree as to cause his death: previous to which, the irritability of stomach was very great, vomiting quantities of black-looking matter repeatedly.

Inflammation of the stomach, I am inclined to think, is the unfortunate combination which, in fatal cases, commonly is the cause of death. In a very few instances, I have observed it in the commencement of the attack, and it was necessary to direct particular attention to this symptom, or combination; for, as there is a possibility of this combination appearing in greater or less degree, so as perhaps to be little heeded, and be allowed to proceed too far, without any precaution being taken to remove it, it is at once accounted for how it may become the cause of death, and confirms the truth of this opinion.

Instances of relapses were few amongst the troops, and none of the lingering symptoms attached to them that have been enumerated in the description of the disease.

Children seemed for some time to be exempt from this disease, but latterly they have also suffered. It was indicated by peevishness, and soreness on being touched; great irritability of stomach; in those who were able to walk, an imperfect manner of using the limbs was observed, causing them frequently to fall. Feverishness was present in all.

There were a few peculiarities noticed in this disease, which entitled it to be considered as a novel and unknown kind of morbid affection. 1st. The extreme violence of the pains in the commencement, and the peculiar sensations they created; 2d, perspiration was not easily excited; 3d, thirst was not much complained of, even in the violence of the fever and in delicate females; 4th, the roseolar eruption above mentioned, and the swelling and tenderness of the hands and feet, was not often observed in the cases in the garrison; 5th, delirium was chiefly confined to delicate females, and aged persons of weak, nervous constitution.

The weather, previous to the appearance of the disease under consideration, and during its continuance, was of a nature unprecedented in severity in the West Indies, at least for very many years.

In the latter end of November, and nearly up to the present period, the weather became extremely boisterous, being nothing but a continuance of heavy rains and high winds; the evenings cold—very cold for this country, so much so that we were obliged to shut our doors and windows on sitting down to dinner; and we found it requisite to cover ourselves with a blanket at night. No Creole constitution could hold out against such weather: they are generally of such a frame that they are not at all capable of undergoing the fatigues and exposure of Europeans who have been a few years in this country. Besides, their mode of living and their habits are also very different from the regiments serving in the country. It was even surprising we escaped as we did; and, although the dress of our men was not altered, which at the time was merely linen trowsers, and which could not guard them very well from the cold damp air of the night, yet we did not suffer in any thing like the way that the natives did. The greater part of our men are young, of strong constitutions, and well fed: it was not to be supposed that they could easily be affected by weather that to them must have been only agreeable.

In treating this anomalous disease, the objects had in view were to lower increased action in the system, and to restore the deranged functions of the vessels of the skin, which might be almost considered the cause of the disease. In accomplishing these purposes, few means were required. The pain of head, and great degree of excitement in young men of stout habits, sometimes required blood-letting, but, generally speaking, it was seldom employed: there was a much better remedy found, which nearly answered both purposes, and that was cathartics. Aloes, colocynth with a combination of calomel, in the following proportions and manner of exhibition, was the usual plan adopted:

> ℞, Extract. Colocynthidis comp., Aloes Socotrinæ, Gummi Resinæ, āā gr. iij.; Hydrargyri Submuriatis gr. xxiv. fiat massa in pilulas duodecim dividend sumantur tres h. s.

On the following morning, the pills were assisted by doses of Infusion of Senna, to which was added a small quantity of neutral salts, or supertartrate of potass. This plan generally required to be repeated once or twice.

When the action of the bowels was thus increased and kept up, the febrile action and violence of the symptoms underwent an almost immediate diminution; particularly the headache, which was the most distressing symptom. Cold water was at the same time applied to the head, by means of folds of linen.

To determine to the skin was another mode of treatment employed, to remove altogether the pains of different parts of the body: this was effected by using the warm bath, and giving small doses of antimonial or James's powder, with a few grains of calomel, three or four times a day; and keeping the body warm, from the commencement of the treatment.

The diet was light and plain. Wine, when it might be advantageously employed, was given.

When pain of stomach was present, which very rarely was the case, and, when it was increased by pressure, its removal was always of the first moment; in doing which, the counter-irritation occasioned by the application of vesicants was a very powerful remedy. Gentle purgative injections at the same time were essentially useful.

With regard to the employment of the sulphate of quinine, I am not able to bear testimony of any power it is supposed to possess in diminishing the violence of the symptoms, or in preventing returns of this disease; and seeing no reason to believe that there existed any morbid consent between the sensorium and deranged impressions of distant parts, I never employed this medicine with the view of defeating its return. —I must confess I used it (the sulphate of quinine) in one or two instances only, before the nature of the disease was exactly declared. It came on in subjects accustomed to frequent attacks of ague, the symptoms of which were chiefly complained of at the beginning. It afterwards turned out that violent pains, such as those that characterise rheumatismus febrilis, were conjoined; therefore cannot positively decide in favor of the sulphate of quinine having any effect in shortening the disease, or in preventing its recurrence when it had apparently gone off.

The disease was very apt to return, or, from having disappeared, was liable to be again excited, if the patients were unguardedly exposed to its causes, which have been stated to be an extraordinary degree of cold and damp in the atmosphere, and the prevalence of high winds, with heavy rain. Under these circumstances, the best security that could be had against its aggravation or recurrence was to defend the body by warm clothing, and confinement to the house, or even to the bedroom.

During the last six weeks we have had rather better weather, but it is still far from being settled, and of late the disease has not appeared in any thing like the frequency it did some time ago.

*St. Christophers; April* 11*th*, 1828.

---

